# Fecal metatranscriptomics of macaques with idiopathic chronic diarrhea reveals altered mucin degradation and fucose utilization

**DOI:** 10.1186/s40168-019-0664-z

**Published:** 2019-03-18

**Authors:** Samuel T. Westreich, Amir Ardeshir, Zeynep Alkan, Mary E. Kable, Ian Korf, Danielle G. Lemay

**Affiliations:** 1 Genome Center, University of California, Davis, California USA; 2 California National Primate Research Center, University of California, Davis, California USA; 3 USDA ARS Western Human Nutrition Research Center, Davis, California USA; 4 Department of Nutrition, University of California, Davis, California USA

**Keywords:** Metatranscriptomics, ICD, Macaque, Microbiome, RNA-seq, Dysbiosis, Pathology, Gut microbiome, Fecal microbiome, Mucin, Gastrointestinal, Diarrhea, Inflammation, Mucosal immunity, Metatranscriptome, Colitis

## Abstract

**Background:**

Idiopathic chronic diarrhea (ICD) is a common cause of morbidity and mortality among juvenile rhesus macaques. Characterized by chronic inflammation of the colon and repeated bouts of diarrhea, ICD is largely unresponsive to medical interventions, including corticosteroid, antiparasitic, and antibiotic treatments. Although ICD is accompanied by large disruptions in the composition of the commensal gut microbiome, no single pathogen has been concretely identified as responsible for the onset and continuation of the disease.

**Results:**

Fecal samples were collected from 12 ICD-diagnosed macaques and 12 age- and sex-matched controls. RNA was extracted for metatranscriptomic analysis of organisms and functional annotations associated with the gut microbiome. Bacterial, fungal, archaeal, protozoan, and macaque (host) transcripts were simultaneously assessed. ICD-afflicted animals were characterized by increased expression of host-derived genes involved in inflammation and increased transcripts from bacterial pathogens such as *Campylobacter* and *Helicobacter* and the protozoan *Trichomonas*. Transcripts associated with known mucin-degrading organisms and mucin-degrading enzymes were elevated in the fecal microbiomes of ICD-afflicted animals. Assessment of colon sections using immunohistochemistry and of the host transcriptome suggests differential fucosylation of mucins between control and ICD-afflicted animals. Interrogation of the metatranscriptome for fucose utilization genes reveals possible mechanisms by which opportunists persist in ICD. *Bacteroides* sp. potentially cross-fed fucose to *Haemophilus* whereas *Campylobacter* expressed a mucosa-associated transcriptome with increased expression of adherence genes.

**Conclusions:**

The simultaneous profiling of bacterial, fungal, archaeal, protozoan, and macaque transcripts from stool samples reveals that ICD of rhesus macaques is associated with increased gene expression by pathogens, increased mucin degradation, and altered fucose utilization. The data suggest that the ICD-afflicted host produces fucosylated mucins that are leveraged by potentially pathogenic microbes as a carbon source or as adhesion sites.

**Electronic supplementary material:**

The online version of this article (10.1186/s40168-019-0664-z) contains supplementary material, which is available to authorized users.

## Background

Rhesus macaques (*Macaca mulatta*) provide a close analog for humans for the study of various conditions, including SIV/AIDS, and are maintained in captive colonies for research purposes [1]. Captive macaque juveniles often develop idiopathic chronic diarrhea (ICD), characterized by repeated episodes of intestinal distress and intestinal inflammation, and lack the presence of known intestinal pathogens detectable by culture-based methods [2, 3]. Successive episodes of ICD cause chronic dehydration. ICD is responsible for the majority of non-medical captive macaque culls [2]. Although pathogenic bacteria have been observed in a minority of cases [4, 5], their presence is not a consistent disease marker. Antibiotics alleviate acute outbreaks, but symptoms re-emerge weeks or months after treatment. Roughly 3–5% of all captive macaques in colonies at the California National Primate Research Center (CNPRC) develop ICD annually [2]. ICD in macaques phenotypically resembles a sub-category of ulcerative colitis in humans in which the ulcers are microscopic. ICD is histologically characterized by lymphocytic, plasmacytic inflammation of lamina propria, goblet cell depletion, crypt hyperplasia, and abscesses [2, 3, 6]. Prior work demonstrated that microbial diversity is decreased in macaques with colitis [7] and that therapeutic helminth infection ameliorates the clinical symptoms of ICD that are associated with the restoration of microbial diversity [3]. However, it is not yet known which features of a microbiome, beyond diversity, are protective against ICD.

Composition of the species dwelling in the gut microbiome varies with diet [8–10], host genetics [11], and a mixture of other genetic and environmental variables [12]. The majority of microbial species in the colon subsists on breakdown products of complex polysaccharides, derived either from the host diet or from host-derived mucins [8, 13, 14]. When adequate fiber is not provided through diet, or as a result of other triggers that are not yet fully understood, the microbiome may dynamically change activity and enzyme production may change when food sources are switched. Increased microbial consumption of host epithelial mucin degrades the barrier matrix between the microbiome and intestinal wall faster than it may be replenished by goblet cells, leading to increased contact between the microbiome and the epithelial wall, greater pathogen susceptibility, and potential invasion by opportunistic pathogens [8, 15–17]. Thus, although inflammation and disease may be triggered by opportunistic pathogens, their ability to gain access to the intestinal wall may be due to a weakening of the mucosal matrix from increased microbial degradation and consumption of the mucin layer [18].

Recent observations of the gut microbiome have focused less on individual species and more on the set of functional niches, each filled by one or more “specialist” capable of preferentially consuming a particular substrate [18]. Evaluation of these activities is possible through application of metagenomic or metatranscriptomic approaches. Although 16S rRNA studies of rhesus macaques have examined the makeup of the macaque gut microbiome [2, 3, 7, 19, 20], there is a lack of understanding about host-microbe and microbe-microbe relationships in ICD. Metatranscriptomics—the genes expressed by all organisms in a sample—can provide observability into which organisms are present and what they are doing [21]. We therefore conducted a metatranscriptomics study of fecal samples from macaques with ICD and their controls to test the hypothesis that ICD is associated with increased gene expression by mucin-degrading bacteria and/or pathogens. We first investigated host-derived transcripts to confirm that inflammation is increased in ICD. We next surveyed the community-wide bacterial metatranscriptome and screened the transcriptomes for specific known bacterial and non-bacterial pathogens. We then probed transcripts specifically from mucin-degrading bacteria and mucin-degrading enzymes. Upon discovering an increase in bacterial fucosidases, we investigated the differential fucosylation of colon sections via immunohistochemistry and further analyzed the metatranscriptome for fucose utilization. The metatranscriptomes suggest two mechanisms by which bacterial pathogens leverage fucosylated mucins in ICD.

## Results

### Host macaque transcripts confirm increased inflammation in ICD

Prior reports of ICD indicate that it is a form of inflammatory bowel disease of the colon [2, 3]. To verify this phenotype in our samples, we investigated the host transcriptome, which is known to include intestinal epithelial cells of the colon that are exfoliated in fecal samples [22]. RNA reads mapped to rhesus macaque (host) proteins were examined to compare host transcript abundance in control and ICD samples. There were 562 host transcripts that were robustly increased in ICD (log2 fold change ≥ 2 relative to control; adjusted *p ≤* 0.05) and 358 host transcripts increased in control animals (log2 fold change ≥ 2 relative to ICD; adjusted *p* ≤ 0.05). Functional enrichment analyses yielded major clusters for transcripts increased in ICD, including “collagen/extracellular matrix(ECM)”, “ECM-receptor interaction/PI3K-AKT signaling,” “inflammatory bowel disease”, and “Toll-like receptor signaling pathway.” Upstream regulator analysis revealed well-known inflammatory regulators interferon gamma (IFNγ), tumor necrosis factor (TNF), interleukin-1 beta (IL1β), and interferon alpha (IFNα) as highly significant (− log(*p*-value) > 30) in ICD animals relative to controls. For host transcripts that were elevated in control relative to ICD, there were no significant upstream regulators.

**Fig. 1:**
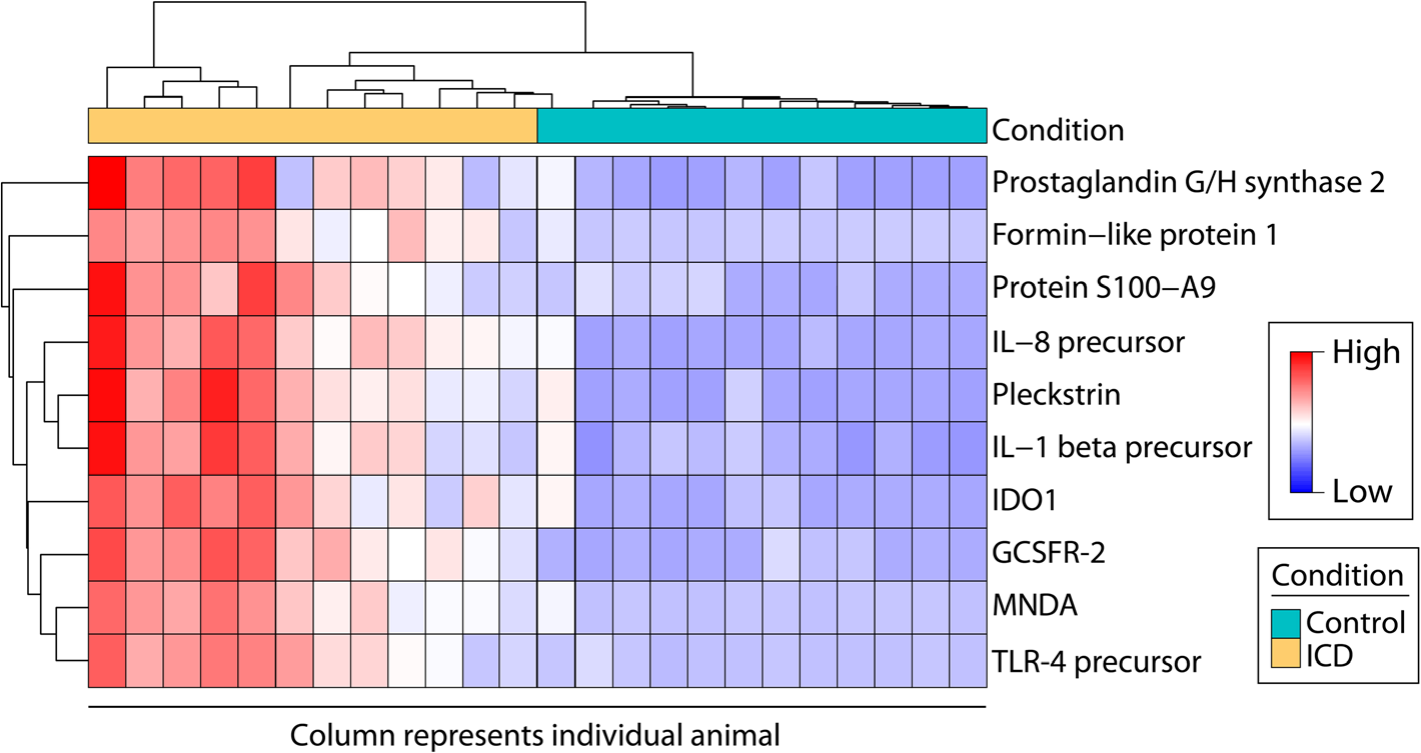
Top ten host transcripts with the greatest fold change in ICD relative to control samples. All transcripts shown were significantly differentially abundant between ICD and control samples. Heatmap color indicates relative log2 fold change per transcript, compared with the average expression level: red indicates higher than average and blue indicates lower than average. Samples (*x*-axis) were hierarchically clustered by expression profile. Colors at the nodes of the top dendrogram indicate control (cyan) or ICD (yellow) samples

Ten host transcripts were 32× higher in ICD than controls (Fig. 1). Prostaglandin G/H synthase 2 (PTGS2, also known as cyclooxygenase-2 or COX-2) is a downstream target of innate immune signaling via the Toll-like receptor 4 (TLR4) signaling pathway. Formin-like protein 1 (FMNL1) is a major component of macrophage podosomes, which is an integrin-based structure that is unique to invasive cells [23]. Protein S100-A9 forms a heterodimer with S100-A8 (also increased in ICD, log2 fold change = 3.80, adjusted *p* = 0.0002). This heterodimer is known as calprotectin, which comprises more than half of the cytosolic protein in neutrophils [24]. Fecal calprotectin is frequently used as a marker of intestinal inflammation [25]. Interleukin-8 precursor (IL-8, also known as CXCL8) is expressed by intestinal epithelial cells to recruit neutrophils from the lamina propria to the epithelium [26]. Pleckstrin (PLEK) is most highly expressed in neutrophils and macrophages (using GeneVisible [27]). Interleukin-1 beta (IL1β) is a pro-inflammatory cytokine that is secreted by activated macrophages and also by colonocytes [28]. Indoleamine 2,3-dioxygenase 1 (IDO1) is an enzyme that is the rate-limiting step in tryptophan catabolism. IDO1 is highly expressed by antigen-presenting cells to reduce tryptophan levels in the local microenvironment, which reduces activated T cell responses and promotes regulatory T cell activity [29]. In addition to antigen-presenting cells, colonocytes also express IDO1 during inflammation [30]. Granulocyte colony-stimulating factor receptor (CSF3R, also known as CD114) is expressed by neutrophils [31]. Myeloid cell nuclear differentiation antigen (MNDA) can be expressed by innate immune cells or intestinal epithelial cells to activate inflammasomes [32]. TLR4 is a receptor expressed by intestinal epithelial cells that recognize bacterial PAMPs, such as lipopolysaccharide [33]. Taken together, these most highly differentially expressed genes suggest that the stool samples from animals with ICD contain cells which express a pro-inflammatory profile.

To determine which host cell types might be differentially present in the stool samples, the expression of cell marker-associated genes were examined. Keratins KRT8, KRT18, KRT19, and KRT20 were all more highly expressed in ICD, confirming that exfoliated colonocytes were present in the stool and more abundant in ICD. Genes CD14, CD44, CD53, and CD97 were more highly expressed in ICD, suggesting that a heterogeneous population of leukocytes may be present in ICD. The high expression of CSF3R, S100-A8, and S100-A9 was supportive of the presence of neutrophils. Thus, the host transcriptome provided some evidence that there were more exfoliated colonocytes and leukocytes in the stool of ICD-afflicted animals compared with controls. Collectively, the host cells in stool from animals with ICD included colonocytes as well as leukocytes, and these cells exhibited a pro-inflammatory transcriptional program.

### Fecal metatranscriptomes had decreased diversity in ICD samples and clustered by phenotype

**Fig. 2:**
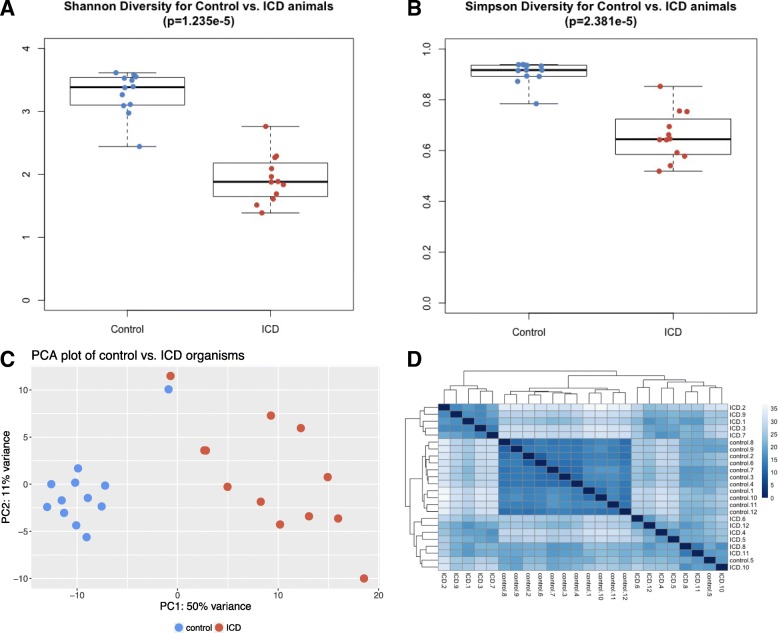
Fecal microbiome diversity differs in ICD compared with controls. Transcript abundances summarized by organism at the genus level were used to calculate Shannon (**a**) and Simpson (**b**) diversity measures. **c** PCA plot of control (red) and ICD (blue) organism metatranscriptome profiles. **d** The heatmap shows the pairwise distances between every pair of whole metatranscriptomes (including pairs of self against self). The darkness of the blue color in the heatmap indicates the Euclidean distance of the variance-stabilized count data from dark blue (smallest distance) to white (largest distance) between each pair of samples. Samples were clustered hierarchically. The dendrograms show the distances between samples with larger branch lengths indicating greater distances

Using all RNA-Seq reads that mapped to bacterial proteins, diversity among the microbial populations was calculated based on transcript abundances, with a 0.1% abundance cutoff threshold for organism identification at the genus level. Comparison of the calculated diversity metrics between samples from control and ICD-afflicted animals showed a significant decrease in diversity in ICD. Using the Shannon diversity calculation, controls had an average diversity score of 3.28, compared with the ICD-associated average diversity score of 1.93 (*p* = 1.245e−5) (Fig. 2a). Similarly, by the Simpson diversity calculation, there was a higher average diversity among control samples when compared with ICD (0.906 vs. 0.656, *p* = 2.380e−5) (Fig. 2b).

Using the same organism transcript abundance measurements with the 0.1% threshold, a principal component analysis (PCA) (Fig. 2c) demonstrated that control and ICD phenotypes clearly segregated, with greater variation seen across ICD samples when compared to the control samples. Hierarchical clustering of whole transcriptomes also showed clustering of control samples (Fig. 2d). However, ICD-associated metatranscriptomes split into two clusters, with some ICD samples (ICD.8, ICD.11, ICD.10) more similar to controls than to other ICD samples. These data suggest greater heterogeneity among the ICD samples. In both the PCA and clustering analyses, control animal 5’s metatranscriptome was more similar to the ICD animals than other controls. Upon re-inspection of life histories, individual macaque control 5 was in the hospital for more days than any other control animal, so it is possible that this animal was not truly a healthy control. However, for the purposes of the remaining analyses, this sample was retained among the controls. In summary, microbial transcriptome diversity was decreased in ICD samples, but heterogeneity between ICD samples was increased.

### Control and ICD samples harbored significant differences in organism-specific gene expression

**Fig. 3:**
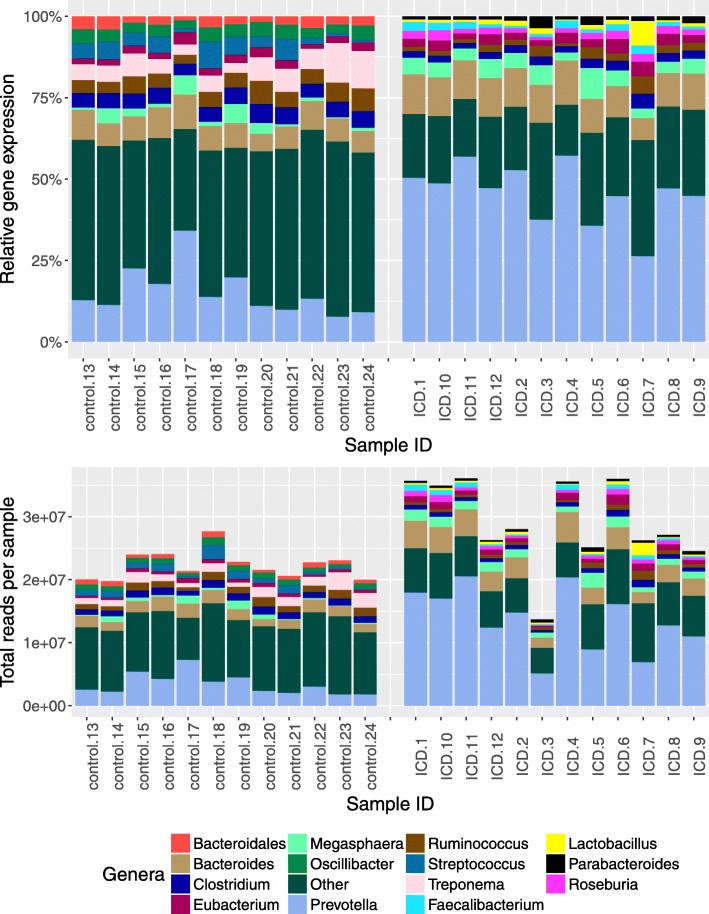
Composition of genera with the highest number of transcripts in stool samples from control and ICD-afflicted macaques. Stacked bar graphs are relative (top) and absolute (bottom) transcript counts per genus in each sample. Only the top ten genera from either control or ICD samples are depicted; all remaining genera are summarized as the “Other” category (dark green)

Using all mRNA reads mapped to bacterial organisms, transcript counts were summarized at the genus level. The genera with the highest transcript abundances in control samples included *Prevotella*, *Bacteroides*, *Treponema*, *Ruminococcus*, *Clostridium*, *Streptococcus*, *Oscillibacter*, *Megasphaera*, *Bacteroidales*, and *Eubacterium* (Fig. 3). The genera with the highest transcript abundances in ICD samples included *Prevotella*, *Bacteroides*, *Megasphaera*, *Eubacterium*, *Clostridium*, *Ruminococcus*, *Roseburia*, *Faecalibacterium*, *Parabacteroides*, and *Lactobacillus*. Although many genera were common between both control and ICD samples, their relative transcript abundances differed; importantly, the “Other” category (all other genera not represented by the ten most abundant) was significantly greater in control samples (Fig. 3). Examination of differential gene expression revealed many differences between ICD and controls with 732 bacterial genera, out of 1511 total detected, showing significantly different expression levels (Additional file 1). Among the genera with the highest transcript abundances, the largest difference was observed in *Prevotella*, which were greatly increased (2.7-log2 fold change, adjusted *p* = 2.68e−16) in the ICD-afflicted animals relative to controls*.* There were also increases in ICD in *Bacteroides* (1.8-log2 fold change, adjusted *p* = 1.09e−13), *Megasphaera* (2.0-log2 fold change, adjusted *p* = 4.82e−6), *Selenomonas* (2.2-log2 fold change, adjusted *p* = 3.56e−14), and *Campylobacter* (2.7-log2 fold change, adjusted *p* = 2.54e−9), whereas there was a decrease in *Treponema* (1.7-log2 fold decrease, adjusted *p* = 7.2e−14). Thus, while many genera expressed genes in both control and ICD samples, their relative expression differed substantially between the two phenotypes, with the largest significant fold changes being among abundant organisms in *Prevotella* and *Campylobacter* genera.

### Community-wide and *Prevotella*-specific transcriptomes suggest differences in nutrient availability between control and ICD samples

**Fig. 4:**
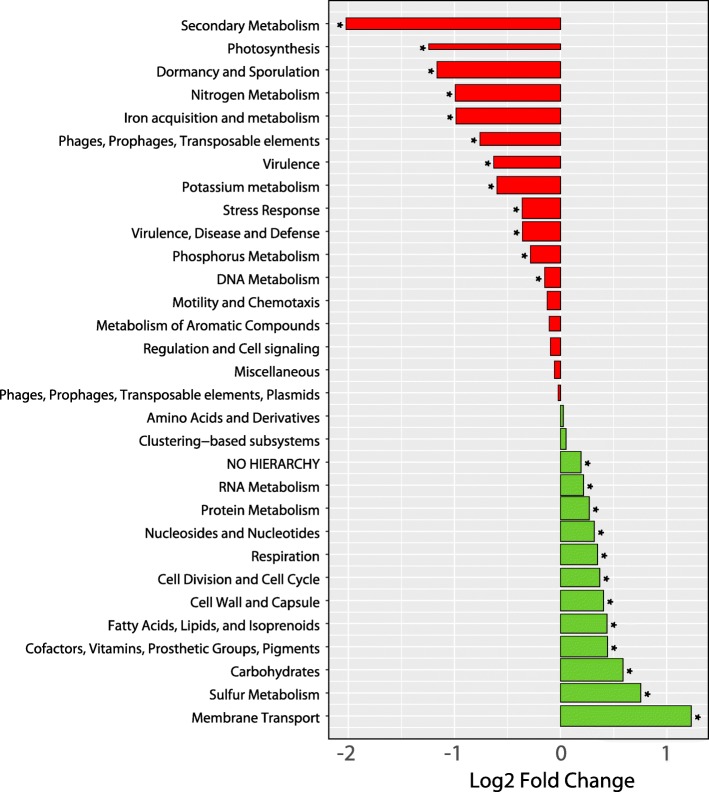
Log2 fold change in ICD cohort relative to controls of all 31 level 1 (top level) categories listed in the SEED Subsystems hierarchy. Functional annotations that are decreased (in ICD relative to controls) are colored in red; increased in ICD are in green. The width of each bar is proportional to the number of transcripts with that annotation. Asterisks indicate significantly differentially expressed categories in ICD relative to controls (*padj* ≤ 0.05)

To further explore microbial community-wide differences in the metatranscriptomes between control and ICD samples, reads were mapped to a database derived from the SEED Subsystems hierarchy [34]. Comparison of the ICD cases to controls revealed changes even at the broadest (level 1) SEED hierarchy categories. The largest increases were in membrane transport (1.23-log2 fold increase, adjusted *p* = 1.76e−29), sulfur metabolism (0.75-log2 fold increase, adjusted *p* = 1.20e−13), and carbohydrate-linked functions (0.58-log2 fold increase, adjusted *p* = 7.72e−23). Notable decreases in ICD-afflicted microbiome functions were seen in the categories of secondary metabolism (2.02-log2 fold decrease, adjusted *p* = 2.82e−8), dormancy and sporulation (1.16-log2 fold decrease, adjusted *p* = 3.59e−7), nitrogen metabolism (0.99-log2 fold decrease, adjusted *p* = 7.1e−6), and iron acquisition and metabolism (0.98-log2 fold decrease, adjusted *p* = 9.84e−13). (Fig. 4). Within the sulfur metabolism category, one significantly increased enzyme was sulfatase (1.8-log2 fold increase, adjusted *p* = 9.92e−7), which may be one of the inflammation-contributing factors [35]. The SEED level 2 annotations “Central carbohydrate metabolism,” “Monosaccharides,” “Polysaccharides,” and “Di- and oligosaccharides” were all increased in ICD (Additional file 2), suggesting ample access to carbon sources [35]. Thus, the overall functional annotation of the bacterial community-wide transcriptome suggests that the fecal microbiome from ICD had better access to nutrients than the control fecal microbiome. This may possibly be due to the faster transit time of diarrhea, which has been shown to alter microbial composition, diversity, and activity [36].

To determine whether the functional differences of the ICD metatranscriptome may be due to faster-growing microbes in the fecal samples as would be typical of the small intestine, we examined annotations related to growth rate. Epithelial cells of the small intestine have a higher turnover rate than cells in the colon [37], and shorter transit times (e.g., diarrhea) have been linked to faster growth of some microbial species [38]. At the community level, “DNA replication” was not significantly different between control and ICD, but the level 3 annotation, “RNA_polymerase_bacterial” was increased in ICD (log2 fold change 0.55, *p* = 0.0001). The concentration of RNA polymerase is known to increase during exponential growth [39]; thus, it is possible that fecal microbes in ICD were growing faster than fecal microbes in controls, which would be consistent with more simple carbohydrate sources reaching the distal colon.

Given that *Prevotella* had greater relative abundance of transcripts in ICD, we investigated the functional annotations of genes specifically expressed by this genus. The largest significant increases in ICD relative to control samples were in the categories of respiration (0.74-log2 fold increase, adjusted *p* = 5.35e−25) and membrane transport (0.68-log2 fold increase, adjusted *p* = 9.98e−24). The largest significant decreases were in the categories of stress response (0.89-log2 fold decrease, adjusted *p* = 1.32e−13), potassium metabolism (0.70-log2 fold decrease, adjusted *p* = 3.03e−5), and phage and transposable elements (0.59-log2 fold decrease, adjusted *p* = 9.82e−7) (Additional file 3). Like the community-wide metatranscriptome, the *Prevotella*-specific transcriptome also had increased “Central carbohydrate metabolism,” “Monosaccharides,” “Polysaccharides,” and “Di- and oligosaccharides” in ICD relative to controls. We next asked whether *Prevotella* had greater “DNA replication” in ICD, but there was actually a slight decrease (− 0.14-log2 fold, adjusted *p* = 0.0041). “RNA_polymerase_bacterial” was statistically unchanged. In summary, the *Prevotella*-specific transcriptome suggests that *Prevotella* in fecal samples of animals with ICD may have had greater access to nutrients without being in an exponential stage of growth.

### Transcripts from bacterial pathogens were more abundant in ICD animals

**Fig. 5:**
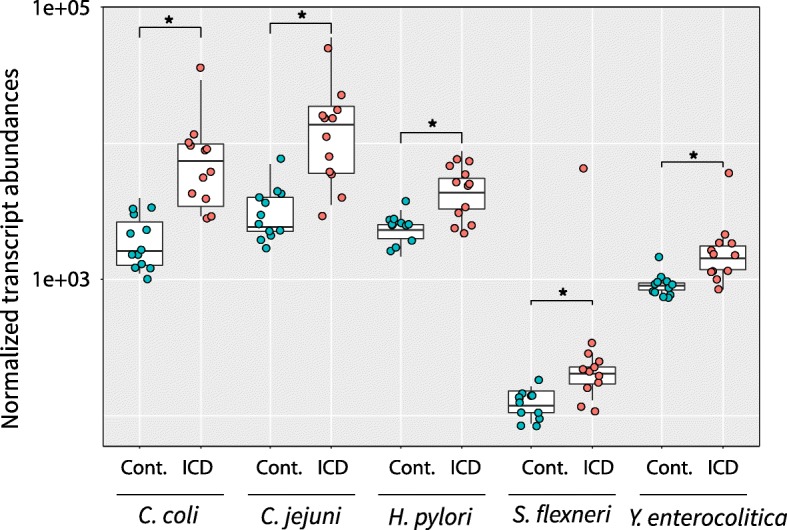
Normalized transcript abundance levels associated with known pathogens in control and ICD samples. Boxplots summarize the distributions of transcript abundances for each pathogen in each control (blue) or ICD (red) samples with individual samples denoted by dots. Stars indicate statistical significance of differences between control and ICD samples (*padj* ≤ 0.05). Normalized transcript abundances are shown on a log scale

Several pathogenic species have been previously linked with ICD [2–4], although none has definitively been proven to be responsible for the condition. We therefore examined the metatranscriptome data for transcripts that mapped to these known pathogens: *Camplyobacter coli* (*C. coli*), *Camplyobacter jejuni* (*C. jejuni*), *Helicobacter pylori* (*H. pylori*), *Shigella flexneri* (*S. flexneri*), and *Yersinia enterocolitica* (*Y. enterocolitica*). Interestingly, some gene expression for all of these pathogens was detectable even in control samples. However, relative transcriptional abundances of all of these known bacterial pathogens were significantly higher in the ICD samples (Fig. 5). The largest differences between control and ICD were in *Campylobacter* species.

### Screening of fungi, protozoa, and archaea yielded potential non-bacterial opportunists

To determine whether non-bacterial opportunists may potentially be active in ICD, fecal metatranscriptome reads were mapped to fungal, protozoan, and archaeal databases. The fungus, *Sordaria macrospora*, had higher transcript abundances in ICD (3.18-log2 fold increase, adjusted *p* = 6.33e−07). Another species from this genus, *Sordaria fimicola*, is known to be present in mammalian dung [40]. *Sordaria fimicola* was not in the reference data set, nor was any other species of *Sordaria*, so it is possible that the active species in macaques was *Sordaria fimicola* due to the limitations of the data. Interestingly, transcripts associated with the known fungal opportunist, *Candida albicans*, were not differentially abundant between control and ICD animals.

Non-human primates are vulnerable to several enteric protozoa, including *Entamoeba histolytica*, *Giardia lamblia*, *Cryptosporidium parvum*, *Cyclospora cayetanensis*, and *Balantidium coli* [41]. *E. histolytica* and *G. lamblia* were among the protozoans with the highest gene expression in this cohort, but their transcripts were not differentially abundant between control and ICD animals. RNA-Seq reads mapping to *Cryptosporidium parvum* were relatively lower in this cohort, compared with *E. histolytica* and *G. lamblia*, and reads mapping to *Cyclospora cayetanensis* were exceedingly rare and likely to be off-target hits. There were no species from the *Balantidium* genus in the data set.

Protozoans with the most abundant transcripts in the fecal samples from the macaques were *Blastocystis* sp. and *Trichomonas vaginalis*. *Blastocystis* sp. was higher in the control animals (1.96-log2 fold decrease, adjusted *p* = 0.03). *T. vaginalis* was higher in the animals with ICD (2.24-log2 fold increase, adjusted *p* = 0.002). *T. vaginalis* was the only species in the reference data set representative of the *Trichomonas* genus, so it is possible that the particular species with increased gene expression in macaques with ICD was not *T. vaginalis*.

Although there are no known pathogens among archaea, some archaeal genera are known to be gut-associated. Among the ten most abundant archaeal genera detected in the fecal samples, six were significantly different between control and ICD animals and all of these were lower in ICD. These include *Methanosarcina* (0.16-log2 fold decrease, adjusted *p* = 0.004), *Methanococcus* (0.16-log2 fold decrease, adjusted *p* = 0.018), *Methanobacterium* (0.13-log2 fold decrease, adjusted *p* = 0.009), *Methanocorpusculum* (0.21-log2 fold decrease, adjusted *p* = 0.044), and *Methanocella* (0.18-log2 fold decrease, adjusted *p* = 0.003). These genera are predominately methane producers. Methane-producing archaea may be depleted in ICD; however, this observation may be confounded by gut transit time, which is reduced by the presence of methane [42]. In summary, ICD was associated with increased gene expression by a fungus (*Sordaria*) and a protozoan (*Trichomonas*) and decreased gene expression by archaeal methanogens.

### Campylobacter expresses mucosa-associated transcripts in ICD

Of all potential bacterial and non-bacterial pathogens (excluding viruses, which are not represented in the metatranscriptome), *Campylobacter* transcripts were the most differentially abundant between ICD and controls. We therefore investigated the functional annotations of genes specifically expressed by this genus. Functional annotation differences between control and ICD animals (Additional file 4) indicate that organisms within this genus experienced more oxidative stress and were more adherent and likely more virulent in ICD relative to control animals. Fermentation, and specifically the abundance of anaerobic respiratory reductases, was reduced in ICD relative to control, suggesting that *Campylobacter* was exposed to a more oxygen-rich environment in the ICD intestine compared with control. Abundance of genes related to oxidative stress and the stringent response ((p)ppGpp_metabolism) were higher in ICD-afflicted animals, whereas genes related to iron acquisition and metabolism were lower in ICD relative to control. This is expected given the inflammatory environment of the colon during ICD. Control of iron acquisition is a common microbial mechanism for reducing exposure to damage via the Fenton reaction.

The oxidative stress that was clearly experienced by *Campylobacter* could potentially be due to a more adherent population in ICD. Genes involved in adhesion were significantly more abundant in ICD animals as were genes for motility and chemotaxis, including flagellum (Additional file 4). Flagellar motility is required for *Campylobacter* colonization [43] and invasion of cultured intestinal epithelial cells [44–46]. This tighter affiliation with the host likely facilitates both increased oxidative stress for the *Campylobacter* present in ICD animals, as described above, and increased virulence. The stringent response, which aids in survival of oxygen stress conditions, has also been shown to be necessary for attachment and invasion of intestinal epithelial cells [47]. Further evidence of a more virulent phenotype in ICD animals was that the pVir plasmid was more nearly four times as abundant (1.8-log2 fold, *p* = 4.66e−04) in ICD animals as in control animals (Additional file 4). Although the definitive role of this plasmid in the virulence of *Campylobacter* has yet to be uncovered, it contains genes with homology to type four secretion system genes in *Helicobacter pylori* and *Agrobacterium tumefaciens*. The mutation of several of these genes affects *C. jejuni* adhesion and invasion in an intestinal epithelial cell culture model [48]. Overall, the *Campylobacter*-specific transcriptome suggests that *Campylobacter* were more closely associated with the mucosa in ICD than in controls.

### Transcripts from mucin-degrading bacteria and mucin-degrading enzymes were increased in ICD

**Fig. 6:**
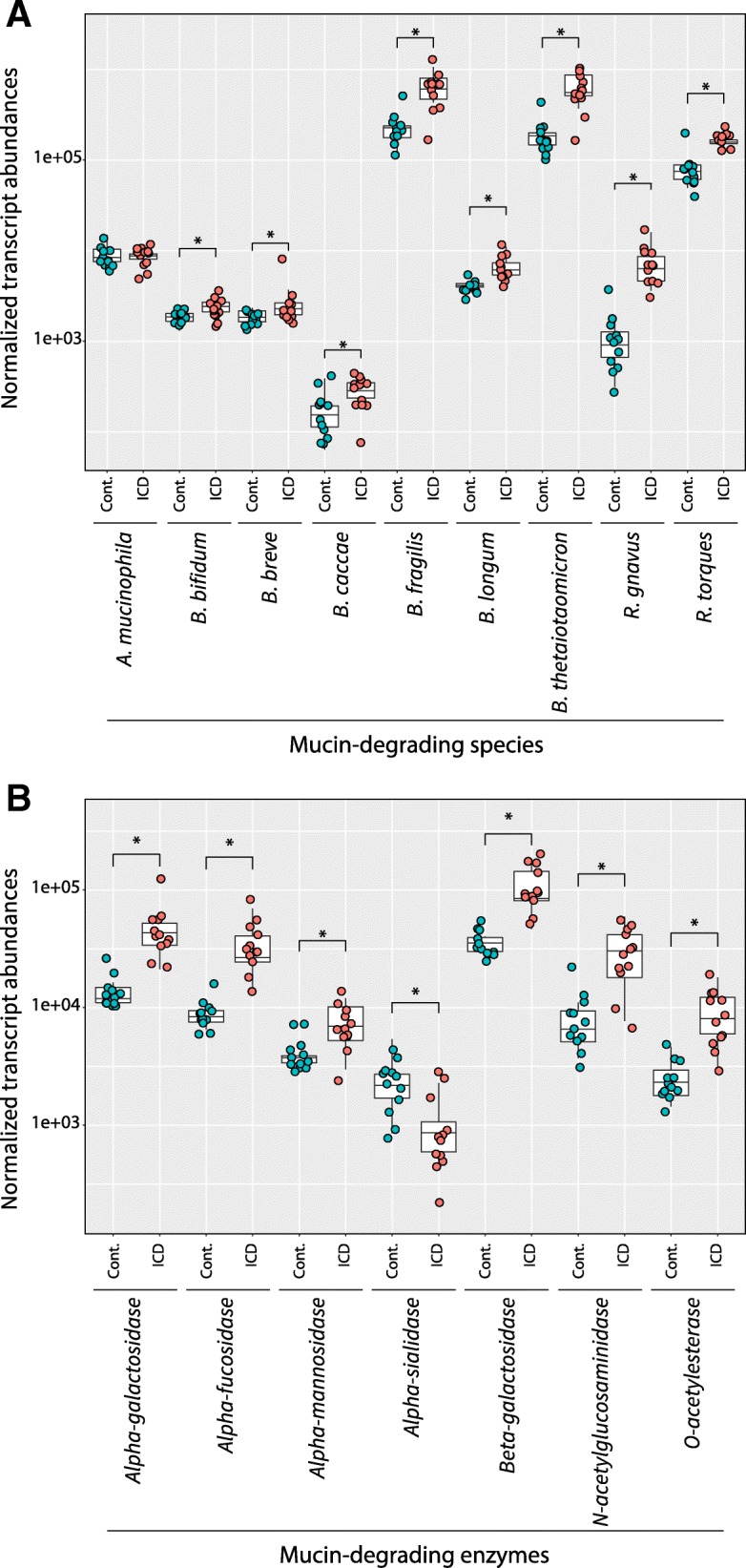
Normalized transcript abundance associated with mucin-degrading species (**a**) and mucin-degrading enzymes (**b**). Boxplots summarize the distributions of transcript abundances for the each species(**a**) or enzyme (**b**) within each group of control (blue) or ICD (pink) samples with individual samples denoted by dots. Stars indicate statistical significance of differences between control and ICD samples (*padj* ≤ 0.05). Normalized transcript abundances (*y*-axis) are shown on a log scale

Having screened the metatranscriptomes for potential pathogens, we next investigated our hypothesis that ICD is associated with increased mucin degradation. First, we subsetted the data to compare abundances of transcripts from bacterial species known to be linked with inflammatory bowel conditions [49], although they are also considered to be commensals. Significantly higher transcript levels were observed in ICD for *Ruminococcus gnavus*, *Ruminococcus torques*, *Bacteroides thetaiotaomicron*, *Bacteroides caccae*, and *Bacteroides fragilis* (Fig. 6a). Abundances of transcripts associated with several *Bifidobacterium* species (*B. bifidum*, *B. breve*, *B. longum*) were slightly, but significantly, higher as well. In short, transcripts associated with all known mucin-degrading gut microbes except for *Akkermasia muciniphilia* were significantly more abundant in the feces of animals with ICD. These organisms are associated with both degradation of the protective mucin layer that forms on the inside of the gut epithelium and with increased presence in human irritable bowel disease [49–51].

Given that mucin-degrading species (Fig. 6a) are also known to be commensals that can thrive on dietary carbohydrates, we next examined the abundances of transcripts arising from the expression of specific enzymes known to be involved in mucin degradation. All of these mucin-degrading enzymes were differentially expressed between ICD and controls (Fig. 6b). Transcript abundances from six of the seven mucin-degrading enzymes—alpha- and beta-galactosidase, alpha-fucosidase, alpha-mannosidase, *N*-acetylglucosamindase, and *O*-acetylesterase—were all higher in ICD. In general, transcripts from both mucin-degrading bacteria and mucin-degrading enzymes were higher in ICD.

### Intestinal cells synthesize fucosylated mucins in ICD

**Fig. 7:**
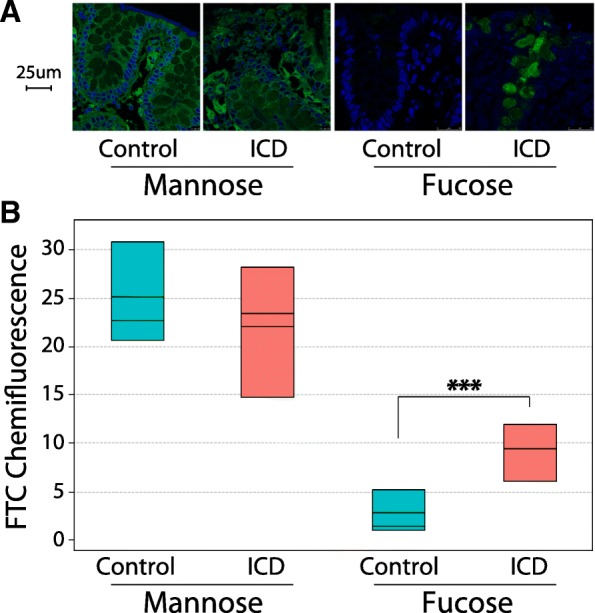
Staining and examination of macaque intestines for mannose and fucose indicates the altered presence of fucose in ICD. Proximal colon sections were stained with 10 μg/mL FITC-labeled *Ulex europaeus* agglutinin 1 or concanavalin A to visualize fucose and mannose, respectively, and counterstained with DAPI. Slides were imaged with a confocal microscope at the same FITC channel voltage setting for each lectin. Five micrographs were analyzed from each animal to calculate a background-corrected crypt mean pixel intensity as described under the “Methods” section. **a** Representative micrographs showing sections from control and ICD animals stained for mannose or fucose. Nuclei stained with DAPI are shown in blue, and mucin sugars are displayed in green. **b** Average crypt pixel intensity of mannose and fucose signal. ***p = 0.008, two-tailed *t* test; *n* = 5 for control, *n* = 6 for ICD animals

Given the differential expression of mucus-degrading enzymes in ICD compared with controls, we hypothesized that control and ICD-afflicted animals may have different mucus composition. Colon sections from control and ICD animals were stained with FITC-conjugated lectins for mannose and fucose (Fig. 7a). All images were selected and analyzed in a blinded manner (animal status unknown). Animals with ICD had markedly higher fucose relative to controls (*p* = 0.008) (Fig. 7b). No changes in intensity were observed for mannose levels between control and ICD animals. These observations suggest that the colons of animals with ICD have greater amounts of fucose than the controls.

Given that transcripts for microbial fucosidases were higher in ICD-associated metatranscriptomes and fucose was more abundant in the ICD-associated colon sections, we asked whether the host cell transcriptome was consistent with what would be expected of increased synthesis of fucosylated mucins. The most highly expressed mucin transcripts were mucin-12 (MUC12), mucin-2 (MUC2), mucin-17 (MUC17), and mucin-13 (MUC13). All of these mucins are known to be expressed by enterocytes in the colon. They were all significantly increased in ICD relative to controls: mucin-12, 2.1-log2 fold change, adjusted *p* = 2.01e−5; mucin-2, 2.3-log2 fold change, adjusted *p* = 3.98e−6; mucin-17, 1.3-log2 fold change, adjusted *p* = 0.005; and mucin-13, 1.6-log2 fold change, adjusted *p* = 1.77e−5. Overall, host fucosyltransferase gene expression was low across all samples. However, the most abundant fucosyltransferase, galactoside 3(4)-l-fucosyltransferase (FUT3), was more highly expressed in ICD (1.5-log2 fold change, adjusted *p* = 0.02) than the controls. Interestingly, the second most abundant fucosyltransferase, galactoside 2-alpha-l-fucosyltransferase 2 (FUT2), which is known to confer secretor status in humans [52], was not differentially expressed between ICD and control. In summary, the immunohistochemistry of colon sections and the examination of the host cell transcriptome suggest that ICD is associated with increased fucosylated mucins.

### Fucose utilization in ICD

Given the increased abundance of transcripts from mucin-degrading enzymes and the increased availability of fucose in the colon of animals with ICD, we next investigated how fucose might be utilized. First, the metatranscriptome was subsetted by the annotation “alpha-l-fucosidase” to determine which microbes were liberating fucose. The top five species expressing this enzyme were all members of the *Bacteroides* genera: *B. fragilis*, *B. xylanisolvens*, *B. vulgatus*, *B. thetaiotaomicron*, and *B. helcogenes*. Expression of alpha-l-fucosidase by these species was significantly increased in ICD relative to controls.

The liberation of fucose from fucosylated mucins by these species is known to increase the amount of available fucose in the lumen [53]. We therefore investigated the expression of “fucose permease” to determine which microbes were importing fucose. The species with the most significantly increased abundance of “fucose permease” transcripts in ICD were *Bacteroides salanitronis*, *Prevotella dentalis*, *Bacteroides fragilis*, *Bacteroides thetaiotaomicron*, *Bacteroides vulgatus*, and *Haemophilus influenzae*. These results suggest that *Bacteroides* could potentially be cross-feeding fucose to *Prevotella* and *Haemophilus* in ICD.

**Fig. 8:**
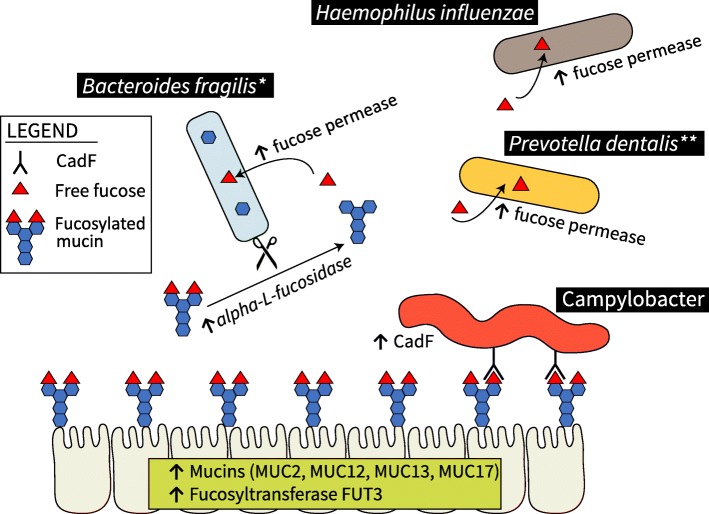
Overview of proposed mechanisms by which fucose is utilized in ICD. Fucose is depicted by red triangles; mucins are depicted by chains of blue hexagons. Host cells (bottom of figure) increase the expression of enterocyte-derived mucins and a fucosyltransferase. Bacteroides species increase the expression of alpha-l-fucosidases, which are secreted enzymes that cleave fucose from host-derived fucosylated mucins, thereby increasing the amount of free fucose in the lumen. Species like *Haemophilus influenzae* and *Prevotella dentalis* increase the expression of fucose permease which enables increased uptake of free fucose. Campylobacter does not increase the expression of fucose permease in ICD, but it does increase the expression of cadF, a protein that enables adherence to fucosylated mucins

An overview of mechanisms proposed by the results in this study is given in Fig. 8. *Bacteroides* sp. is known to stimulate host production of fucosylated mucins [54]. We found that enterocyte mucins and fucosyltransferase-3 transcripts were elevated in the host transcriptome. Immunohistochemistry of colon sections also illustrated the much higher presence of fucose in ICD relative to controls. Multiple *Bacteroides* sp. in ICD expressed more alpha-l-fucosidase, which liberates fucose from fucosylated mucins in the lumen. *Bacteroides* sp. as well as other microbes, such as *Prevotella* and *Haemophilus*, expressed fucose permeases which would have enabled them to import fucose, which likely improves their persistence in ICD. Interestingly, *Campylobacter* did not express more transcripts that give rise to proteins which import fucose; instead, it expressed more transcripts for adherence proteins, like cadF, that would have enabled it to adhere to fucosylated mucins.

## Discussion

Previous work showed that the gut microbiome of ICD had reduced diversity [55], but the salient features of that microbiome remained unknown. In the current study, we confirmed the reduced diversity at the transcriptional level and identified numerous novel features, such as relatively higher expression of mucin-degrading enzymes and of transcripts from known pathogens, such as *Campylobacter*, and altered fucose utilization.

Some of the novel features of ICD could be due to the capturing of fast-transit material from the small intestine, as might be expected of a diarrhea sample. For example, transcriptional features such as decreased diversity, lower stress, better access to simple sugars, and lower methane production are all consistent with what might be expected of a small intestine microbiome [56] or a faster transit time [36, 38]. However, mucin foraging is a primary feature in the colon; thus, our findings of increased gene expression by mucin-degrading bacteria, increased expression of mucin-degrading enzymes, increased expression of mucin and fucosylation by host cells, and increased fucose utilization by specific bacteria suggest these strong signals of the metatranscriptome in ICD do not arise purely from the proximal intestine. Furthermore, the colon sections, which were extracted from the same part of the colon in both ICD and control animals, showed differential presence of fucose.

Transcript matches to organisms at the genus level revealed domination of the ICD-associated fecal microbiome by multiple *Prevotella* sp. and a smaller but still significant increase by *Bacteroides*. Elevated *Prevotella* presence is also noted in other dysbiotic conditions, such as HIV [57] and intestinal and systemic inflammation in humans [58]. It has additionally been shown, using a dextran sodium sulfate (DSS)-treated mouse model, that the human intestinal *Prevotella* isolate, *P. copri*, is capable of exacerbating externally induced inflammation [59]. On the other hand, *Prevotella* is known to be increased in the microbiomes of healthy individuals habitually consuming a long-term vegetarian diet [60]. Therefore, it is possible that increased transcripts from *Prevotella* in ICD are merely a marker of increased carbohydrate availability. Relative to control samples, central carbohydrate metabolism was highly increased in the *Prevotella*-specific transcriptome during ICD. Annotations also suggest that *Prevotella* was not under oxidative stress. *Prevotella* may have filled nutritional niches for food carbohydrates after antibiotic exposure, which is common for macaques with repeated episodes of diarrhea.

*Prevotella* and *Bacteroides* are often considered to be antagonistic; gut microbiome communities are usually abundant in *Prevotella* or *Bacteroides* but not both [61]. One explanation for the prevalence of both genera in ICD could be related to cross-feeding. The annotation “Sialic_acid_Metabolism” was increased in the *Prevotella*-specific transcriptome during ICD, but not “sialidase” (Additional file 3). Meanwhile, *B. thetaiotaomicron* has sialidases and can free sialic acid, but lacks the genetic capability to consume free sialic acid [62]. It is therefore possible that *Bacteroides* cross-feeds *Prevotella* in ICD.

One of the drawbacks of our metatranscriptomic approach is that we were unable to distinguish the difference between many transcripts from fewer bacteria compared with fewer transcripts from many bacteria. For this purpose, metagenomics combined with metatranscriptomics would have yielded a more powerful data set and should be considered for future studies. Nevertheless, the metatranscriptomic analysis was immensely useful to reveal microbe-microbe and host-microbe interactions.

We investigated the hypothesis that ICD was associated with pathogens that were missed via traditional culture-based methods. We observed increased gene expression by numerous bacterial pathogens, primarily *Campylobacter* and *Helicobacter*. Observed increases in gene expression by *Campylobacter* and *Helicobacter* in the ICD samples are consistent with previous observations of increased abundance in gut dysbiosis [4, 19, 63]. *Helicobacter* was previously positively linked with macaque ICD [4], and *Campylobacteraceae* was more commonly found in macaques with colitis [7]. Interestingly, our data suggests that these organisms are present in both ICD and control samples, but their transcripts are more abundant in ICD.

We also found elevated gene expression by a fungal genus and a protozoan genus. Increased transcripts were observed from the *Sordaria* genus of fungi in ICD; this genus is commonly found in the intestine of herbivores [40]. Annotation against a protozoan-specific database revealed elevated gene expression by *Trichomonas*, a common protozoan flagellate present in macaques both with and without active ICD [64]. It is unclear whether these are innocent bystanders or causative agents.

Perhaps just as important as the positive associations of potential pathogens with ICD is what can be safely ruled out as causative agents. We did not find evidence of increased gene expression by any of the usual suspects among protozoans for enteric disease in rhesus macaques. These include *Entamoeba histolytica*, *Giardia lamblia*, *Cryptosporidium parvum*, *Cyclospora cayetanensis*, and *Balantidium coli* [41]. It is noteworthy that we did not see an expansion of the bacterial family *Enterobacteriaceae*, which has been observed in other dysbiotic intestinal environments. This suggests that chronic ICD is not indicative of an environment with a high level of oxygen-related stressors [65].

Ordinarily, the colonic epithelium is protected against pathogens by layers of mucus. Biopsies from humans with ulcerative colitis have revealed dysfunctional mucus that can be penetrated by bacteria [66]. We investigated the hypothesis that ICD is associated with increased mucin degradation. We found relatively more transcripts from mucin-degrading bacteria and mucin-degrading enyzmes in ICD than in controls. It was previously postulated that disruption of the mucus barrier leads to persistent irritation and inflammation of the gut epithelium during ulcerative colitis in humans and ICD in macaques [2, 3, 50]. Further support for this theory comes from the success of helminthic therapy, in which the addition of helminth whipworms stimulates new mucin production, reduces pro-inflammatory markers, and alleviates ICD symptoms [3]. The relative increase in transcripts associated with known mucin-degrading species posits that these microbes may be at least in part responsible for continued degradation of the protective mucin layer, although further investigation is needed to determine the magnitude of their influence and the level of mucosal degradation.

In the current study, both host transcript abundances and the immunohistochemistry of colon sections provided evidence of increased fucosylation of mucins in ICD. Although fucosylation is usually beneficial to the host in support of commensal microbes, it can also be leveraged by pathogens, particularly after insults with antibiotics have removed commensals [67]. Pickard and Chervonsky [67] summarize mechanisms used by pathogens to exploit fucose that falls into two categories: use as a carbon source or use as an adhesion site. In our data, we found evidence of both mechanisms that were used by different pathogens. *Haemophilus influenzae* had increased fucose permease transcripts in ICD. While *Haemophilus* did not express a fucosidase transcript, *Bacteroides* sp. increased transcripts for alpha-l-fucosidases in ICD; these are secreted enzymes that are known to increase the amount of free fucose in the lumen [53], thus making fucose available to species like *H. influenzae*. Evidence for the second mechanism was seen with *Campylobacter*, which did not have increased expression of fucose permease transcripts but did have elevated transcripts for cadF, a protein that enables *Campylobacter* to adhere to fucosylated glycans. It is known that *Campylobacter* chemotaxes toward fucose and is able to bind fucosylated mucins. The overall transcriptional profile of *Campylobacter* in ICD further supports the finding that it adheres to the mucus because its transcriptional profile is consistent with what would classically be expected of a pathogen in close contact with the slightly oxygenated intestinal epithelial layer: increased oxidative stress and increased secretion systems.

Tropini et al. [68] postulate that the spatial reprogramming of commensals is an important feature of chronic inflammatory bowel disease, which is associated with inappropriate immune activation of mucus-degrading commensals mislocalized to the mucosa. Our data suggest that this is also true of ICD in macaques. We suggest a model in which commensals like *Bacteroides* stimulate the host to produce fucosylated mucins, which then sustain those commensals as well as other cross-feeding species. The fucosylated mucins also serve as adherence sites for microbes like *Campylobacter*, which has the capacity to stimulate inflammation and continue the viscous cycle.

## Conclusions

Although the cause of ICD remains unclear, the current study provides several clues. The data confirm several previous observations, such as decreased microbial diversity and increased host inflammation among animals with ICD. More importantly, the data dramatically extend prior observations to include new associations with ICD: increased transcripts associated with *Prevotella*, with known pathogens such as *Campylobacter*, *Helicobacter*, and *Trichomonas*, and with mucin-degrading bacteria. Relatively higher expression of microbial genes in ICD that give rise to mucin-degrading enzymes is suggestive of a dysfunctional microbiome that degrades the mucus barrier instead of catabolizing dietary polysaccharides. During ICD, the host appears to produce fucosylated mucins that are leveraged by microbes as a carbon source or as adhesion sites, enabling the sustained presence of potentially pathogenic species.

## Methods

### Collection of macaque fecal samples

Fecal samples were collected from a cohort of 12 healthy controls and 12 ICD-diagnosed juvenile macaques. ICD animals were diagnosed clinically being non-responsive to previous antibiotic treatment and having had three consecutive negative culture results for enteric pathogenic bacteria (*Shigella flexneri*, *Yersinia enterocolitica*, *Campylobacter jejuni*, and *Salmonella*) and negative IFA results for parasites (*Cryptosporidium* and *Giardia*) within the 30 days prior to sample collection. In addition to age, ICD and control animals were approximately matched for early-life environment and most recent environment: all animals had spent the first 90 days of life in an outdoor environment and the last 30 days before sample collection in an indoor environment. None of the animals received antibiotics within 30 days of sample collection.

To collect stool samples, all animals were intermittently pair-housed (ICDs.

and controls separately) indoors. In the afternoon, a clean cage pan (metal tray) was placed under the cage after separating the paired animals. Stool samples were collected from the cage pan of each animal the next morning. When possible, stool was collected within approximately 15 min after the morning meal. Stool samples were collected using disposable sterile transfer pipets into 15-mL conical tubes containing approximately 5 volumes of RNAlater and archived in a − 80 °C freezer after collection.

### RNA extraction from macaque fecal samples

Each RNAlater-preserved stool sample was vortexed and homogenized after thawing for 10 min. Approximately 0.6 g of stool slurry was incubated with lysis buffer and periodically vortexed. The sample was homogenized using a bead beater with 0.1-mL beads, followed by QIAshredder treatment. Extraction was performed on the homogenized sample using the Qiagen RNeasy RNA Isolation kit, with additional Turbo DNAse treatment to remove lingering DNA contamination. RNA concentration and integrity were verified using NanoDrop and Bioanalyzer traces.

### Metatranscriptome sequencing

For each sample, RNA-Seq libraries were prepared using > 2 μg total extracted RNA at the DNA Sequencing Core of the UC Davis Genome Center. All extracted RNA samples were first ribodepleted using the RiboZero Magnetic Gold Kit (Epidemiology version), catalog number MRZE706. The Illumina TruSeq protocol, without poly(A) selection, was used to prepare the RNA-Seq libraries. The 24 samples were run on 6 lanes of Illumina HiSeq 3000 at 100 bp, paired-end, with indexing to allocate ~ 25% of the lane to each sample metatranscriptome. This approach yielded approximately 95 million raw reads per sample.

### Annotation and analysis of metatranscriptome reads

The raw metatranscriptome files were preprocessed, annotated, and analyzed using a modified version of the SAMSA2 pipeline [21, 69]. Preprocessing involved removal of low-quality sequences and adaptor contamination with Trimmomatic [70] and paired-end alignment with PEAR [71], removing approximately 40% of raw reads. Cleaned sequences were annotated by DIAMOND [72] with an *e* value cutoff of 0.001; reads were mapped against NCBI’s most recent RefSeq non-redundant protein release [73] and against SEED Subsystems [34]; approximately 45% of cleaned reads met quality score cutoffs to receive an annotation. Specific versions of the SEED and NCBI databases used in this project are available via a bioshare link on GitHub (https://github.com/transcript/macaqueICD, see the “Databases Used” section of the README). On average, roughly 25 million transcripts per sample received an annotation against the RefSeq NR Bacteria database. The minimum number of per-sample annotations was 11 million; the maximum was 36.5 million. For downstream analysis, the resulting annotation files were aggregated and merged using custom Python and R scripting [21]. Statistical computations were performed using the DESeq2 package for R [74]. Individual organism and functional identifications were drawn from NCBI’s RefSeq database and the SEED [34, 73]. Subsequent subsetting of data to profile specific organisms in regard to their transcript abundances was performed using Python scripting included with the SAMSA2 pipeline [21, 69].

### Functional enrichment analyses

The Database for Annotation, Visualization and Integrated Discovery (DAVID) v6.8 was used to conduct gene set enrichment analyses [75]. For each differentially expressed gene set, statistically significant genes were sorted by fold change, and only those with log2 fold change ≥ 2 and adjusted *p ≤* 0.05 were used for enrichment analyses. For each analysis, *Macaca mulatta* was selected as the background gene list and the Benjamini and Hochberg adjusted *p* value ≤ 0.05 was taken to be significant. Upstream regulator analysis was conducted on the same differentially expressed gene sets using Ingenuity Pathways Analysis [76].

### Identification and annotation of host organism reads

A DIAMOND custom database was created using all rhesus macaque protein sequences, accessed through NCBI’s RefSeq repository (release 102, created 22 December 2015). Metatranscriptome raw reads were annotated against this host sequence database, with an *e* value cutoff of 0.001, to identify macaque RNA transcripts. There were between 1.5 million and 2 million host-associated annotations per individual macaque.

### Statistical analyses

All statistical analyses of the metatranscriptomics data were conducted using scripts adapted from SAMSA2 [21, 69]. R scripts used in the current project are available at https://github.com/transcript/macaqueICD/tree/master/R_scripts. Specifically, PCA and clustering analyses were conducted using make_DESeq_PCA.R and make_DESeq_heatmap.R, respectively. Differential abundance testing of transcripts was conducted using DESeq2 [74]. By default, DESeq2 computes a Benjamini and Hochberg adjusted *p* value (*padj*) to correct for multiple hypothesis testing. We used *padj* ≤ 0.05 as statistically significant.

### Histology staining and analysis

Proximal colon tissues were collected at the time of necropsy and immediately submerged in 10% neutral buffered formalin after collection for 72 h or until processed. To acquire formalin-fixed paraffin-embedded (FFPE) tissues, formalin-fixed samples were submerged in a new jar of formalin at 37 °C for 1 h, then incubated in a series of 70% ethanol (EtOH) at 37 °C for 30 min three times, 95% EtOH at 37 °C for 1 h twice, 100% EtOH at 37 °C for 1 h three times, toluene at 37 °C for 1 h twice, and paraffin at 62 °C for 1 h twice, and embedded during the last stage in paraffin at 62 °C for 1 h. Tissue blocks were cut by Anatomic Pathology Service at UC Davis Veterinary Medicine. Five-micron-thick FFPE proximal colon sections were de-paraffinized and rehydrated with xylene and a graded series of alcohol. Sections were blocked with 10% BSA in PBS for 1 h, washed with PBS, and then incubated with 10 μg/mL FITC-labeled lectin (*Ulex europaeus* agglutinin 1 and concanavalin A for fucose and mannose, respectively; Vector Laboratories, Burlingame, CA) in PBS for 1 h in a dark, humidified container at room temperature. Slides were washed with PBS, mounted with ProLong Diamond Antifade Mountant with DAPI (ThermoFisher Scientific) and a 170-μm-thick glass coverslip, and cured for 24 h at room temperature. Slides were imaged at UC Davis Health Sciences District Advanced Imaging Facility with a Leica TCS SP8 STED 3X confocal microscope at the same FITC channel voltage setting. Five micrographs were selected and analyzed from each animal while blinded to animals’ status as ICD or control. Average FITC channel pixel intensity was determined from a polygon selection of single crypt and a blank area from the same image with ImageJ 1.47v software to calculate a background-corrected crypt mean pixel intensity.

## Additional files

Additional file 1: Macaque_bacteria_genus_DESeq_results. This tab-separated file contains the DESeq2 output of differential bacterial gene expression at the genus level between ICD and control samples, with adjusted *p* values. The controlMean refers to mean counts of the control samples. ExperimentalMean refers to mean counts of the ICD samples. Log2 fold changes refer to ICD relative to control. The *p* values are adjusted for multiple hypothesis testing. (TSV 149 kb)

Additional file 2: All_bacteria_Subsystems_DESeq_results. This Excel spreadsheet contains the differential expression by SEED Subsystem hierarchies between ICD and control samples for community-wide metatranscriptomes. The first tab contains a description of all hierarchies included in the analyses. The remaining tabs contain results corresponding to the level 1, level 2, and level 3 hierarchies. The controlMean refers to mean counts of the control samples. ExperimentalMean refers to mean counts of the ICD samples. Log2 fold changes refer to ICD relative to control. The *p* values are adjusted for multiple hypothesis testing. (XLSX 444 kb)

Additional file 3: Prevotella_Subsystems_DESeq_results. This Excel spreadsheet contains the differential expression by SEED Subsystem hierarchies between ICD and control samples for *Prevotella*-specific transcriptome (only those reads mapping to *Prevotella*). The first tab contains a description of all hierarchies included in the analyses. The remaining tabs contain results corresponding to the level 1, level 2, and level 3 hierarchies. The controlMean refers to mean counts of the control samples. ExperimentalMean refers to mean counts of the ICD samples. Log2 fold changes refer to ICD relative to control. The *p* values are adjusted for multiple hypothesis testing. (XLSX 365 kb)

Additional file 4: Campylobacter_Subsystems_DESeq_results. This Excel spreadsheet contains the differential expression by SEED Subsystems hierarchies between ICD and control samples for *Campylobacter*-specific transcriptome (only those reads mapping to *Campylobacter*). The first tab contains a description of all hierarchies included in the analyses. The remaining tabs contain results corresponding to the level 1, level 2, and level 3 hierarchies. The controlMean refers to mean counts of the control samples. ExperimentalMean refers to mean counts of the ICD samples. Log2 fold changes refer to ICD relative to control. The *p* values are adjusted for multiple hypothesis testing. (XLSX 409 kb)
